# ﻿Two new species of *Centroptilum* Eaton, 1869 from North Africa (Ephemeroptera, Baetidae)

**DOI:** 10.3897/zookeys.1131.91017

**Published:** 2022-11-22

**Authors:** Thomas Kaltenbach, Laurent Vuataz, Boudjéma Samraoui, Sara El Yaagoubi, Majida El Alami, Jean-Luc Gattolliat

**Affiliations:** 1 Museum of Zoology, Palais de Rumine, Place Riponne 6, CH-1005 Lausanne, Switzerland Museum of Zoology Lausanne Switzerland; 2 University of Lausanne (UNIL), Department of Ecology and Evolution, CH-1015 Lausanne, Switzerland University of Lausanne Lausanne Switzerland; 3 Laboratoire de Conservation des Zones Humides, Université 8 Mai 1945 Guelma, Guelma, Algeria Université 8 Mai 1945 Guelma Guelma Algeria; 4 Department of Biology, University Badji Mokhtar Annaba, Annaba, Algeria University Badji Mokhtar Annaba Annaba Algeria; 5 Laboratoire Ecologie, Systématique, Conservation de la Biodiversité (LESCB), Unité de Recherche Labellisée CNRST N°18, Université Abdelmalek Essaâdi, Faculté des Sciences, Département de Biologie, B.P.2121 93002 Tétouan, Morocco Université Abdelmalek Essaâdi Tétouan Morocco

**Keywords:** Algeria, biogeography, COI, mayflies, Morocco, Palearctic, taxonomy

## Abstract

Based on recently collected larvae from Algeria and Morocco, the species delimitation within the genus *Centroptilum* Eaton, 1869 in that region is validated. Two new species are described and illustrated, one from north-eastern Algeria, and one from North Morocco, using an integrated approach with morphological and molecular evidence. A table summarising the morphological differences between the new species and *Centroptilumluteolum* (Müller, 1776) from Central Europe is provided. Further, molecular evidence for additional undescribed species of *Centroptilum* in other regions of the West Palearctic is provided and discussed.

## ﻿Introduction

Thomas (1998) provided a provisional checklist of the mayflies from the Maghreb including 69 species: 41 from Morocco, 50 from Algeria, and 29 from Tunisia. This checklist included 17 species of Baetidae, nine additional species of this family needed to be confirmed. During the last two decades, important improvements were made in the knowledge of North African mayflies. A few new species of Baetidae, Leptophlebiidae, Heptageniidae, and Prosopistomatidae were described from Tunisia, Algeria, and Morocco (Soldán et al. 2005; Zrelli et al. 2011; Benhadji et al. 2018; Kechemir et al. 2020; Dambri et al. 2022; El Alami et al. 2022b), and new reports were provided for countries or basins, especially for Tunisia (Zrelli et al. 2011, 2012, 2016), East and West Algeria (Benhadji et al. 2020; Samraoui et al. 2021a, b), and Morocco (Khadri et al. 2017; Mabrouki et al. 2017; El Alami et al. 2022a; Zerrouk et al. 2021). A few species were morphologically revised including in some cases the description of previously unknown stages (Soldán et al. 2005; Zrelli et al. 2012; Godunko et al. 2018). However, the status of several species needs confirmation, especially concerning widely distributed Palearctic species originally described from Central Europe. An integrative approach, based on multiple evidence like morphological, molecular, ecological, and biogeographical data, should be widely used to solve this riddle. Among these problematic cases are the various reports of *Centroptilumluteolum* (Müller, 1776) from Algeria, Morocco, and Tunisia that need to be confirmed.

The genus *Centroptilum* Eaton, 1869 originally encompassed only the two species distributed in Europe and North America. It was, at that time, mainly defined by imaginal characters, adults being mostly similar to *Cloeon* Leach, 1815, but different by the presence of narrow hindwings with a long costal process. The generic concept was rapidly broadened to encompass all Baetidae with single intercalary veins and presence of hindwings. Species from all biogeographical regions, including Australasia, were assigned to this genus with the highest diversity in the Afrotropical and Nearctic regions. The generic concept was step by step circumscribed mainly by excluding the Afrotropical species and creating new genera to accommodate them (Gillies 1990; Lugo-Ortiz and McCafferty 1998). In the Maghreb, the species *Centroptilumdimorphicum* (Soldán & Thomas, 1985) was assigned to the Afrotropical genus *Cheleocloeon* Wuillot & Gillies, 1993 (Lugo-Ortiz and McCafferty 1997). Finally, the concept of *Centroptilum* was restricted to the type species *C.luteolum* (Kluge 2012, 2016). All species previously attributed to *Centroptilum* were either assigned to other genera such as *Anafroptilum*, *Neocloeon*, and *Cloeon* or considered as *Incertae sedis* (*Centroptilumcollendum* Harker, 1957 and *Centroptilumelongatum* Suter, 1986 from Australia) or species *inquirenda* (*Centroptilumpirinense* Ikonomov, 1962 from the Balkans). The history and concept of the genus *Centroptilum* were recently summarised in detail by Martynov et al. (2022). In the same article, the authors described a new species from the South Caucasus. They provided a table with all reliable characters to securely separate the species within *Centroptilum*. They also gave genetic evidence that the European populations of *C.luteolum* are most probably diphyletic and correspond to two putative species.

The genus *Centroptilum* was reported from the whole Maghreb. In Tunisia, the genus seems to be extremely rare as Boumaïza and Thomas (1995) only reported a single larva in their extensive survey of the country; they also considered it to be the most sensitive species to ionic concentration. In Algeria, the genus has a very limited distribution as it was recently only collected in the El Kala basin (Samraoui et al. 2021a); it seems to be absent from surrounding basins in East Algeria and other parts of the country (Benhadji et al. 2020; Samraoui et al. 2021b). Its distribution is also limited in Morocco as it was only collected in the northern part of the country (El Alami et al. 2022a). As already previously stated (Samraoui et al. 2021a; El Alami et al. 2022a), the genus *Centroptilum* needs to be revised in North Africa. In the present study, we use recently collected specimens from north-eastern Algeria and North Morocco to validate the species delimitation, and to describe two new species; we use an integrative approaches combining morphological and molecular evidence.

## ﻿Materials and methods

The specimens from Algeria were collected between 2018 and 2020 by BS, and the specimens from Morocco in 2014 and 2021 by MEA and collaborators. Comparative material from Switzerland was collected by André Wagner (MZL). The larvae were preserved in 70%–96% ethanol.

The dissection of larvae was done in Cellosolve (2-Ethoxyethanol) with subsequent mounting on slides with Euparal liquid, using an Olympus SZX7 stereomicroscope.

Drawings were made using an Olympus BX43 microscope. To facilitate the determination of the new species and the comparison of important structures with other species, we partly used a combination of dorsal and ventral aspects in the same drawing (see Kaltenbach et al. 2020: fig. 1c).

**Figure 1.**
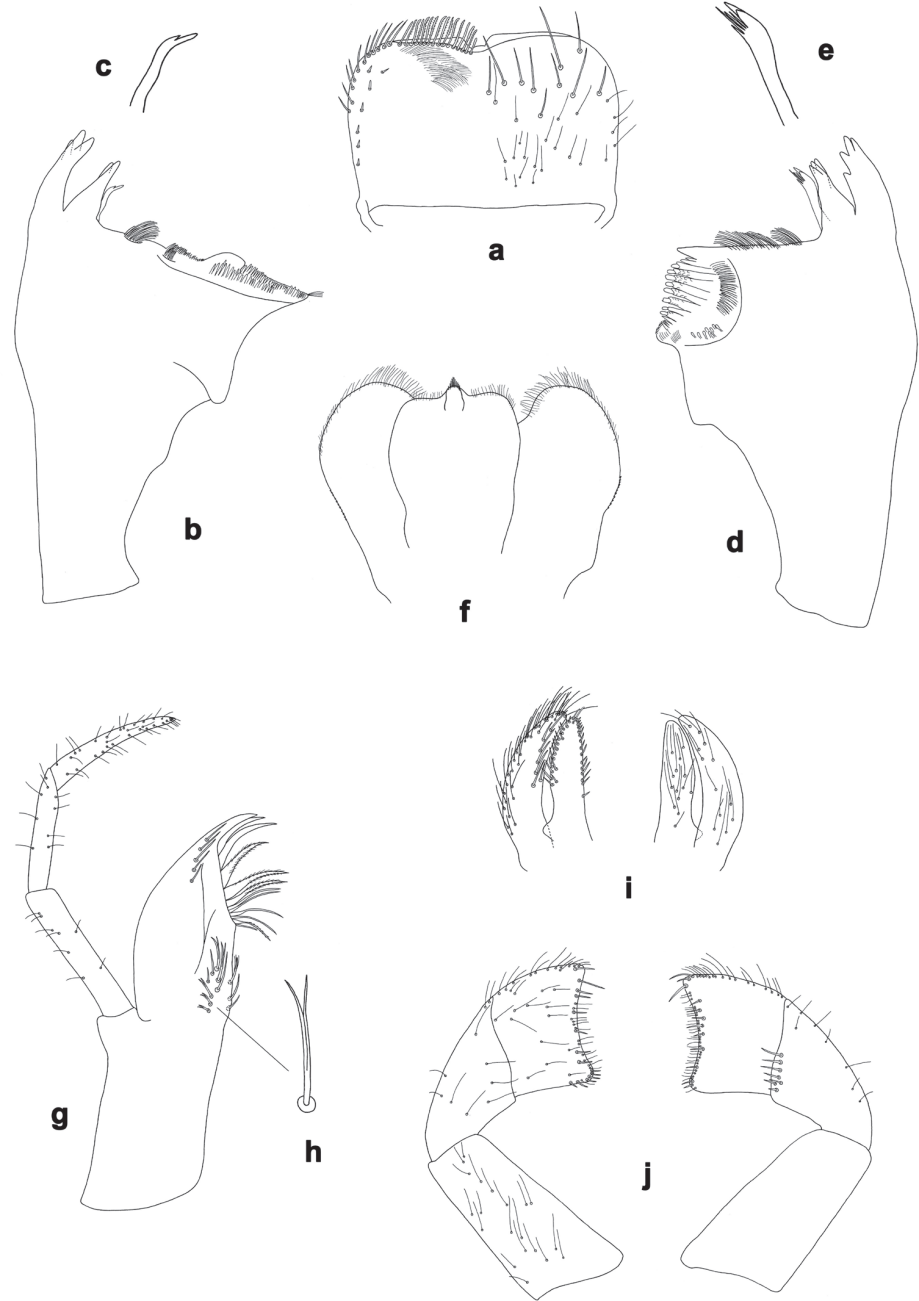
*Centroptilumsamraouii* sp. nov., larva morphology **a** labrum (left: ventral view; right: dorsal view) **b** right mandible **c** right prostheca **d** left mandible **e** left prostheca **f** hypopharynx and superlinguae **g** maxilla **h** seta, ventrolateral **i** glossa and paraglossa (left: ventral view; right: dorsal view) **j** labial palp (left: ventral view; right: dorsal view).

Photographs of larvae were taken using a Canon EOS 6D camera and processed with Adobe Photoshop Lightroom (http://www.adobe.com) and Helicon Focus v. 5.3 (http://www.heliconsoft.com). Photographs of body parts of the larvae were taken with an Olympus BX51 microscope equipped with an Olympus SC50 camera and processed with Olympus (recently Evident) software Stream Basic v. 1.3. All pictures were subsequently enhanced with Adobe Photoshop Elements 13.

Distribution maps were generated with SimpleMappr (https://simplemappr.net, Shorthouse 2010). The GPS coordinates of the sample locations are given in Table 1. The terminology follows Hubbard (1995) and Kluge (2004). Table 2 of this study was partly developed based on Martynov et al. (2022: table II).

**Table 1.** Examined and sequenced specimens.

Species	Country	Location	Coordinates	Specimen catalogue #	GenBank #(CO1)	GenSeq Nomenclature
*Centroptilumsamraouii* sp. nov.	Algeria	Louar inf.	36°37'03"N, 08°22'49"E	GBIFCH00763735	OP113123	genseq-2 COI
		Guitna sup.	36°36'42"N, 08°21'19"E	GBIFCH00895417	OP113124	genseq-2 COI
				GBIFCH00895418	OP113125	genseq-2 COI
				GBIFCH00654969	OP113126	genseq-2 COI
		Guitna inf.	36°37'05"N, 08°20'47"E	GBIFCH00975621	n/a	n/a
*Centroptilumalamiae* sp. nov.	Morocco	Oued Kelâa	35°14'32"N, 05°10'10"W	GBIFCH00980875	OP113127	genseq-2 COI
				GBIFCH00980876	OP113128	genseq-2 COI
		Oued Jnane Niche	35°15'29"N, 04°52'42"W	GBIFCH00975647	n/a	n/a
*Centroptilum* sp.	Iran	Javarem	36°13'43"N, 52°54'32"E	GBIFCH00763741	OP113129	genseq-4 COI

For the molecular part of the study, we first downloaded all *Centroptilum* cytochrome oxidase subunit 1 (COI) sequences available on GenBank as on 13.04.2022 using a custom script, resulting in 99 records. We then manually removed all sequences from specimens collected outside the Western Palearctic, resulting in 34 European sequences for further analyses. We also examined the sequences available on the BOLDSYSTEMS data portal as on 13.04.2022, but excluded all sequences shared with GenBank, those from specimens collected outside the Western Palearctic, and one sequence that did not blast with *Centroptilum* (i.e., most probably resulting from a misidentification or a contamination). As a result, no additional sequence could be obtained. We also included three sequences from the European mayfly FREDIE project (unpublished; https://wp.fredie.eu/). Finally, seven specimens were newly sequenced for this study (Table 1; the nomenclature of gene sequences follows Chakrabarty et al. (2013)), for a total of 44 *Centroptilum* sequences in our molecular data set. The DNA of the sequenced specimens was extracted using non-destructive methods allowing subsequent morphological analysis (see Vuataz et al. 2011 for details). We amplified a 658 bp fragment of the COI gene using the primers LCO 1490 and HCO 2198 (Folmer et al. 1994, see Kaltenbach and Gattolliat 2020 for details). Sequencing was done with Sanger’s method (Sanger et al. 1977). Forward and reverse sequencing reads were assembled and edited in CodonCode Aligner 10.0.2 (Codon-Code Corporation, Dedham, MA), and aligned using MAFFT (Katoh et al. 2019) with default settings as implemented in Jalview 2.11.2.2 (Waterhouse et al. 2009). The best evolutionary model (HKY+ Γ +I) was selected following the second-order Akaike information criterion (AICc; Hurvich and Tsai 1989) implemented in JModelTest 2.1.10 (Darriba et al. 2012) with seven substitution schemes and all other parameters set to default. In order to accommodate different substitution rates among COI codon positions, we analysed our data set in two partitions, one with first and second codon positions and one with third positions (1 + 2, 3). Bayesian inference (**BI**) gene tree reconstruction was conducted in MrBayes 3.2.7a (Ronquist et al. 2012). Two independent analyses of four MCMC chains run for five million generations with trees sampled every 1'000 generations were implemented, and 500'000 generations were discarded as a burn in after visually verifying run stationarity and convergence in Tracer 1.7.2 (Rambaut et al. 2018). One representative of four species belonging to the same subfamily as *Centroptilum* (i.e., Cloeoninae sensu Bauernfeind and Soldán 2012) were used as outgroup. The consensus tree was visualised and edited in iTOL 6.5.7 (Letunic and Bork 2021).

To explore COI evolutionary divergence and compare it to our morphological identifications, we applied three single-locus species delimitation methods to our CO1 data set: the distance-based **ASAP** (Assemble Species by Automatic Partitioning; Puillandre et al. 2020), the tree-based **GMYC** (General Mixed Yule-Coalescent; Pons et al. 2006; Fujisawa and Barraclough 2013), and **mPTP** (multi-rate Poisson Tree Processes; Kapli et al. 2017) approaches. The ASAP method, which is an improvement of the widely used **ABGD** (Automatic Barcode Gap Discovery; Puillandre et al. 2012) approach, has the advantage of providing a score that designates the most likely number of hypothetical species. The GMYC model, which requires a time-calibrated ultrametric tree as input, implements a Maximum Likelihood (ML) approach that defines a threshold separating the branches modelled under speciation events (Yule process) from those described by allele neutral coalescence. The **mPTP** approach, which is a multi-rate extension of the PTP (Poisson Tree Processes; Zhang et al. 2013), also exploits intra- and interspecies phylogenetic differences, but with the advantage of directly using the number of substitutions from a phylogenetic tree, eliminating the need for time calibration.

ASAP was applied to our COI alignment using the ASAP webserver available at https://bioinfo.mnhn.fr/abi/public/asap/asapweb.html, computing the genetic distances under the Kimura 2-parameter substitution model (K2P; Kimura 1980) with all other settings set to default. Input BI ultra-metric tree for GMYC was generated in BEAST 1.10.4. (Suchard et al. 2018). To avoid potential biases in threshold estimation, the outgroups were removed, and identical CO1 haplotypes were pruned (see Talavera et al. 2013) using Collapsetypes 4.6 (Chesters 2013). Input BEAST file was created in BEAUTi (Suchard et al. 2018), implementing the best model of evolution and the partition scheme specified above, and selecting a relaxed molecular clock (uncorrelated lognormal) model, a coalescent (constant size) prior (see Monaghan et al. 2009) and a UPGMA starting tree. Two independent MCMC chains were run for 50 million generations, sampling trees every 1'000 generations. Run convergence was visually verified in Tracer and the independent log and tree files were combined using LogCombiner 1.10.4 (Suchard et al. 2018) after discarding 10% of the trees as burn-in. The maximum clade credibility tree, generated in TreeAnnotator 1.10.4 (Suchard et al. 2018) with all options set to default, was used as input for GMYC, which was run in R 4.2.0 (R Core Team 2022) using the SPLITS package 1.0-20 (Ezard et al. 2009). We favoured the single-threshold version of the GMYC model because it was shown to outperform the multiple-threshold version (Fujisawa and Barraclough 2013). Input ML tree for mPTP was generated in RAxML-NG 1.1.0 (Kozlov et al. 2019) from our CO1 alignment (outgroup included), selecting the all-in-one (ML search + bootstrapping) option and MRE-based bootstrap convergence criterion. The best model of evolution and the partition scheme specified above, as well as 50 random and 50 parsimony starting trees were implemented. mPTP was conducted on the web service available at https://mptp.h-its.org. Finally, the number of parsimony-informative sites and the mean COI genetic distances between and within species were calculated in MegaX (Kumar et al. 2018; Stecher et al. 2020) under the K2P model.

### ﻿Abbreviations:

**MZL** Musée de Zoologie Lausanne (Switzerland);

**LESCB** Laboratoire Ecologie, Systématique, Conservation de la Biodiversité, Tétouan (Morocco).

## ﻿Results

### ﻿Taxonomy

#### ﻿ Centroptilum samraouii

Taxon classificationAnimaliaEphemeropteraBaetidae

Kaltenbach, Vuataz & Gattolliat sp. nov.

05D708EB-910D-5746-98C7-DA1192F054E8

https://zoobank.org/C04FC672-92F6-4E55-8B48-FB4D5BDD93BD

**Figure 2.**
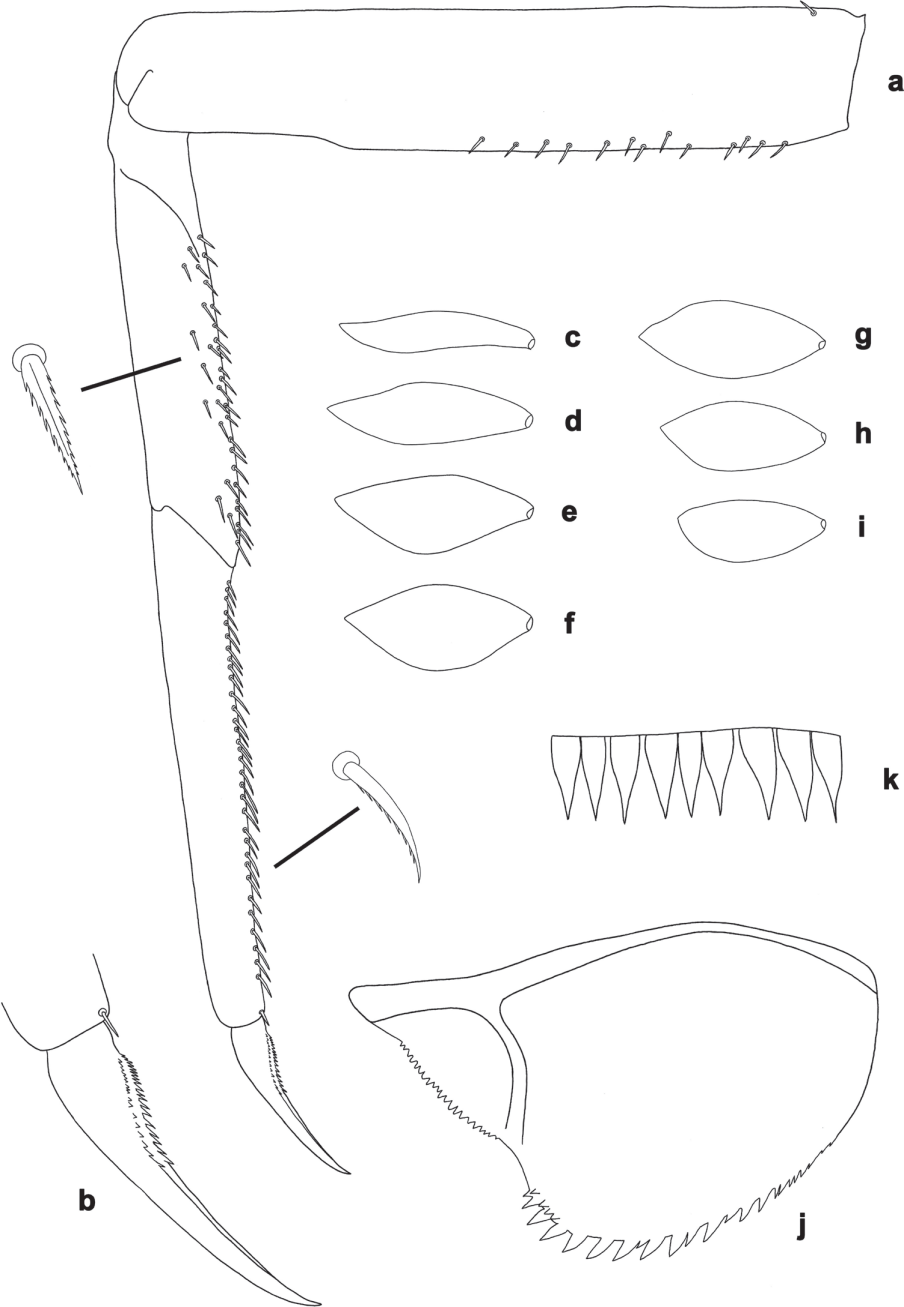
*Centroptilumsamraouii* sp. nov., larva morphology **a** foreleg **b** fore claw **c** tergalius I **d** tergalius II **e** tergalius III **f** tergalius IV **g** tergalius V **h** tergalius VI **i** tergalius VII **j** paraproct **k** caudalii, spines on posterior margin of segments.

**Figure 3.**
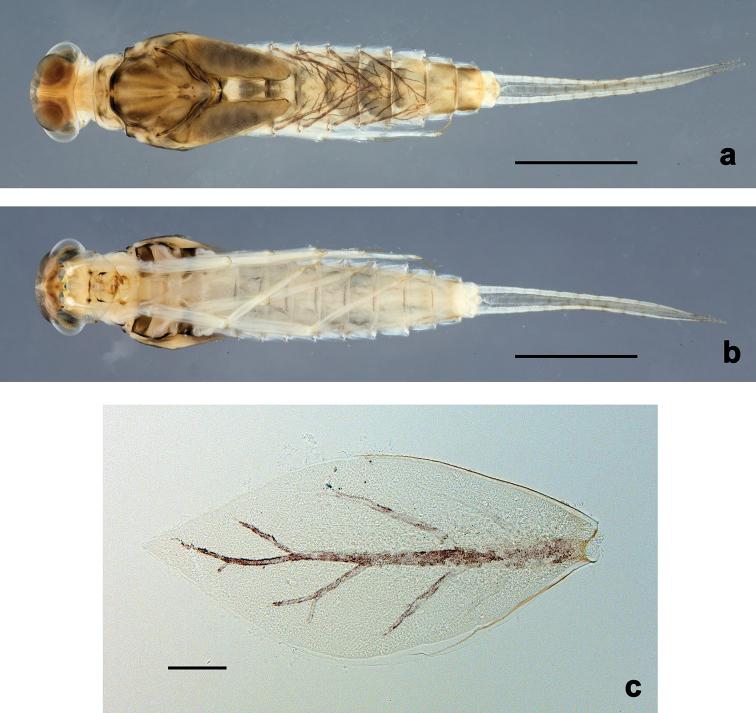
*Centroptilumsamraouii* sp. nov., larva **a** habitus, dorsal view **b** habitus, ventral view **c** tergalius IV. Scale bars: 1 mm (**a, b**); 0.1 mm (**c**).

**Figure 4.**
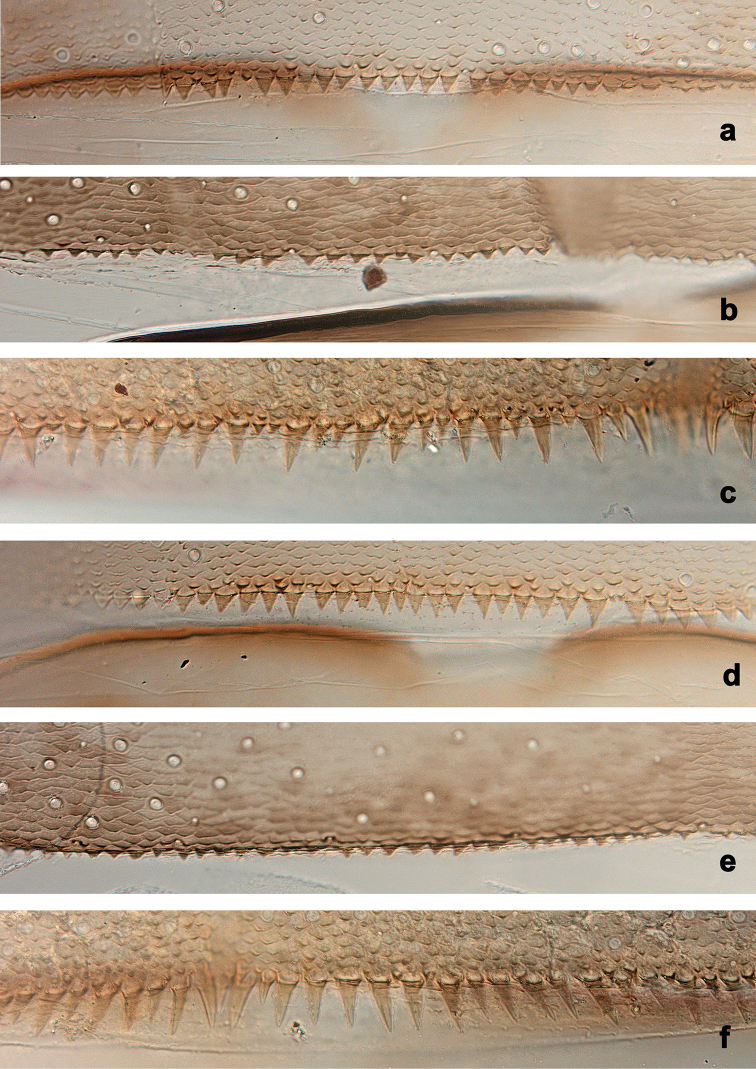
Larvae, posterior margins of terga. *Centroptilumsamraouii* sp. nov. **a** tergum III **d** tergum IV; *Centroptilumalamiae* sp. nov. **b** tergum III **e** tergum IV; *Centroptilumluteolum*: **c** tergum III **f** tergum IV.

**Figure 5.**
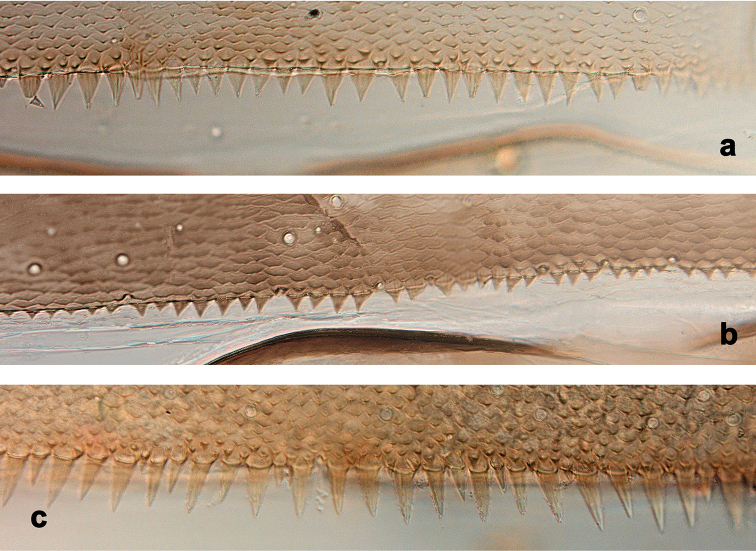
Larvae, posterior margins of terga VII **a***Centroptilumsamraouii* sp. nov. **b***Centroptilumalamiae* sp. nov. **c***Centroptilumluteolum*.

**Figure 6.**
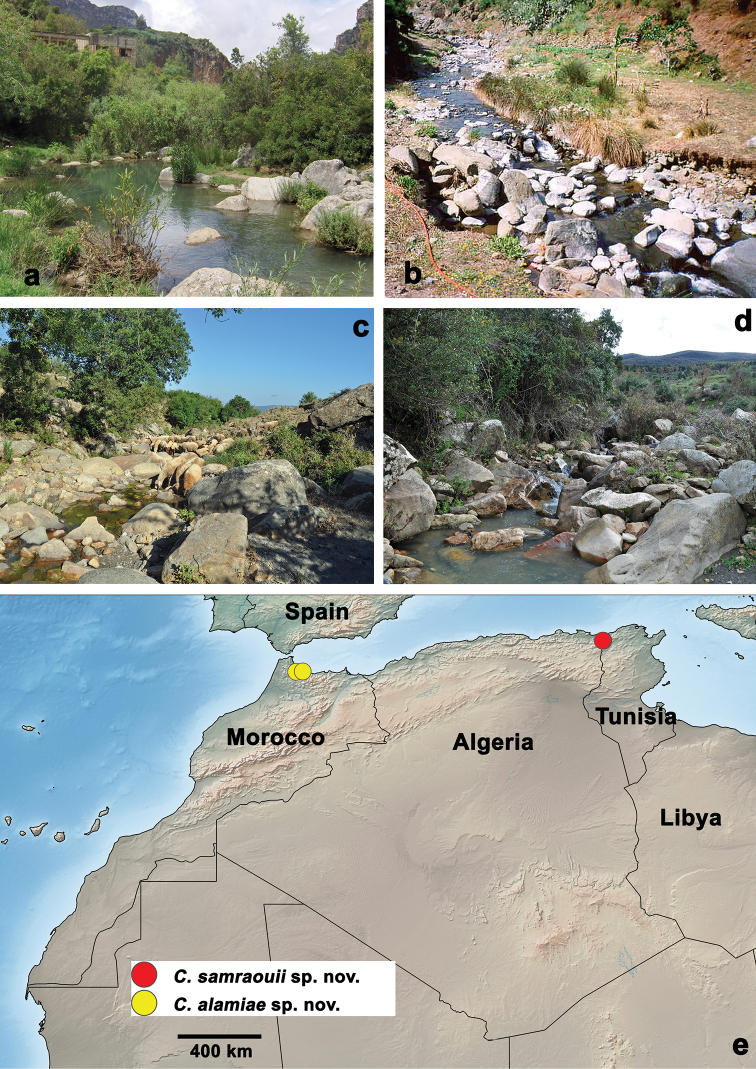
Habitats and distribution of the new species **a, b***Centroptilumalamiae* sp. nov., habitats **a** Oued Kelâa (type locality) **b** Oued Jnane Niche **c, d***Centroptilumsamraouii* sp. nov., habitats **c** Guitna sup. (type locality) **d** Louar inf. **e** distribution map.

Figs 1 , 2 , 3 , 4a, d , 5a , 6

##### Differential diagnosis to other species of *Centroptilum*.

**Larva.** Following combination of characters: A) labrum with anterior margin nearly straight; ratio width vs. length ca. 1.6× (Fig. 1a); B) maxillary palp ca. 1.9× as long as galea-lacinia, segment III apically pointed; segment III ca. 1.3× as long as segment II (Fig. 1g); C) inner distal margin of labial palp segment III concave (Fig. 1j); D) dorsal margin of fore femur with occasional short, spine-like setae (Fig. 2a); E) fore tarsus slightly longer than tibia (1.1×; Fig. 2a) F) claw with two rows of denticles, each with ca. 20 small to minute denticles (Fig. 2b); G) paraproct with 17–23 pointed spines, plus some additional submarginal spines (Fig. 2j).

##### Description.

**Larva** (Figs 1–3, 4a, d, 5a). Body length 3.8–4.2 mm. Cerci: ca. 2/3 of body length. Paracercus: nearly as long as cerci. Antennae reaching apex of fore protoptera.

***Colouration*** (Fig. 3a, b). Head, thorax and abdomen dorsally brown, with dark grey-brown pattern as in Fig. 3a. Head and thorax ventrally brown, with dark grey-brown lateral marks on thorax (Fig. 3b). Abdomen ventrally light brown. Legs light brown, apex of femur and claw darker. Caudalii ecru, brown annulated.

***Labrum*** (Fig. 1a). Rectangular, width ca. 1.6× maximum length. Distal margin with broad, angulated, medial emargination. Anterior margin nearly straight. Dorsal surface scattered with long, medium and short, simple setae; setae not arranged in a submarginal arc. Ventrally with marginal row of setae composed of anterolateral long, simple, pointed setae and medial long, apically blunt, pectinate setae; ventral surface with ca. seven short, stout setae near lateral and anterolateral margin.

***Right mandible*** (Fig. 1b, c). Incisor and kinetodontium separated. Incisor with three denticles; kinetodontium with two denticles. Prostheca stick-like, distally with two denticles. Margin between prostheca and mola almost straight, with two tufts of long setae. Tuft of setae at apex of mola present.

***Left mandible*** (Fig. 1d, e). Incisor and kinetodontium separated. Incisor with four denticles; kinetodontium with three denticles. Prostheca stick-like, distally denticulate. Margin between prostheca and mola straight, with large brush-like tuft of long setae. Subtriangular process short, on level of area between prostheca and mola. Tuft of setae at apex of mola absent.

***Hypopharynx and superlinguae*** (Fig. 1f). Lingua as long as superlinguae. Lingua longer than broad; distal half laterally not expanded; distal margin with short, fine setae, tuft of stout setae short. Superlinguae distally rounded; lateral margins rounded; fine, short to long, simple setae along distal margin.

***Maxilla*** (Fig. 1g, h). Galea-lacinia ventrally with 3–5 simple, apical setae under canines. Canines long and slender. With three denti-setae, distal denti-seta canine-like, middle and proximal denti-setae slender, bifid and pectinate. Medially with one pectinate, spine-like seta and two simple, spine-like setae (dorsolateral insertions); and ca. eight long setae with bifurcated tips (bifurcation often difficult to see; ventrolateral insertions). Maxillary palp 3-segmented, ca. 1.9× as long as length of galea-lacinia; palp segment III ca. 1.3× length of segment II; setae on maxillary palp fine, simple, scattered over surface of segments I, II, and III; apex of last segment pointed.

***Labium*** (Fig. 1i, j). Glossa nearly as broad and slightly shorter than paraglossa; inner and outer margins with many short, spine-like setae; apex with two medium, robust setae; dorsal surface with long, fine, simple, scattered setae. Paraglossa curved inward; ventrally with many long setae along outer lateral and apical margin, and row of long, stout, pointed, simple setae along inner lateral margin; dorsal surface with long, fine, simple, scattered setae. Labial palp 3-segmented. Segment III nearly trapezoidal with rounded distal corners, distal margin concave; outer lateral margin with short to medium, fine, simple setae, distal margin with short, spine-like and short, fine, simple setae; ventral surface with medium, fine, simple, scattered setae. Segment II with medium, fine, simple, scattered setae along outer lateral margin and on ventral surface; dorsally with 5–7 short, spine-like setae along distal margin. Segment I with medium, fine, simple setae scattered on ventral surface.

***Hind protoptera*** well developed.

***Foreleg*** (Fig. 2a, b) very slender. Ratio of foreleg segments 1.6:1.0:1.1:0.4. *Femur*. Length ca. 5× maximum width. Dorsal margin with occasional short, spine-like setae. Apex slightly rounded. Short, stout, pointed setae scattered along ventral margin; femoral patch absent. *Tibia*. Dorsal margin bare. Ventral margin with row of short, curved, spine-like setae and additional stout, pointed setae along margin. Anterior surface scattered with few stout, pointed, and partly serrate setae along ventral margin. Patellatibial suture present in basal ¼ area. *Tarsus*. Dorsal margin bare. Ventral margin with dense row of short, curved, serrate, spine-like setae. *Claw* with two rows of 17–20 minute denticles each, in basal ca. 1/3 area, increasing in size distally; subapical setae absent.

***Terga*** (Figs 4a, d, 5a). Posterior margin of terga: I smooth, without spines; II with rudimentary spines; III with small, triangular spines; IV–IX with triangular spines.

***Sterna.*** Posterior margin of sterna I–VI smooth, without spines. Posterior margin of sterna VII–VIII with small, triangular spines.

***Tergalii*** (Figs 2c–i, 3c). Present on segments I–VII. Costal margins with minute denticles and short, fine, simple setae, anal margins almost smooth. Tracheae extending from main trunk to inner and outer margins. Tergalius I as long as length of segments II–IV combined; tergalius IV as long as length of segments V and VI combined; tergalius VII as long as length of segments VIII and IX combined.

***Paraproct*** (Fig. 2j). With 17–23 pointed marginal spines of different size, and some additional spines in second row. Cercotractor with minute, irregular, marginal spines.

***Caudalii*** (Fig. 2k). Spines at posterior margins of segments elongated triangular with long points.

**Subimago.** Judging from subimaginal tarsomeres developing under cuticle of last instar female larvae, all tarsomeres of all legs of female subimago have pointed microlepids on surface (see Kluge 2022).

**Imago.** Unknown.

##### Etymology.

Dedicated to Prof. Boudjéma Samraoui, committed researcher on aquatic insects in Algeria, and collector of the new species; in recognition to his substantial contribution to the knowledge of the ecology and distribution of Algerian mayflies.

##### Biological aspects.

*Centroptilumsamraouii* sp. nov. occupies the headwaters of steep, narrow and intermittent streams (Fig. 6c, d; Samraoui et al. 2021b, c).

##### Distribution

(Fig. 6e). Algeria.

##### Type-material.

***Holotype*.** Algeria • larva; Guitna sup., Ghora; 36°36'42"N, 08°21'19"E; 22.01.2020; leg. B. Samraoui; on slides; GBIFCH00592552, GBIFCH00592551, GBIFCH00592622; MZL. ***Paratypes*.** Algeria • 2 larvae; Guitna sup., Ghora; 36°36'42"N, 08°21'19"E; 05.11.2019; leg. B. Samraoui; on slides; GBIFCH00895417, GBIFCH00895418; MZL • 3 larvae; Guitna sup.; 36°36'42"N, 08°21'19"E; 09.10.2019; leg. B. Samraoui; on slide; GBIFCH00592553; 2 in alcohol; GBIFCH00975620, GBIFCH00975623; MZL • larva; Louar inf., Ghora; 36°37'03"N, 08°22'49"E; 05.11.2019; leg. B. Samraoui; on slide; GBIFCH00592555; MZL • larva; Algeria; Guitna inf.; 07.11.2018; leg. B. Samraoui; in alcohol; GBIFCH00975621; MZL.

#### ﻿ Centroptilum alamiae

Taxon classificationAnimaliaEphemeropteraBaetidae

Kaltenbach, Vuataz & Gattolliat sp. nov.

10F69263-64BA-5F4A-BC8F-E4177CB8C73B

https://zoobank.org/0468CE29-CFF8-4DF7-ABB9-562D1C9B099F

**Figure 7.**
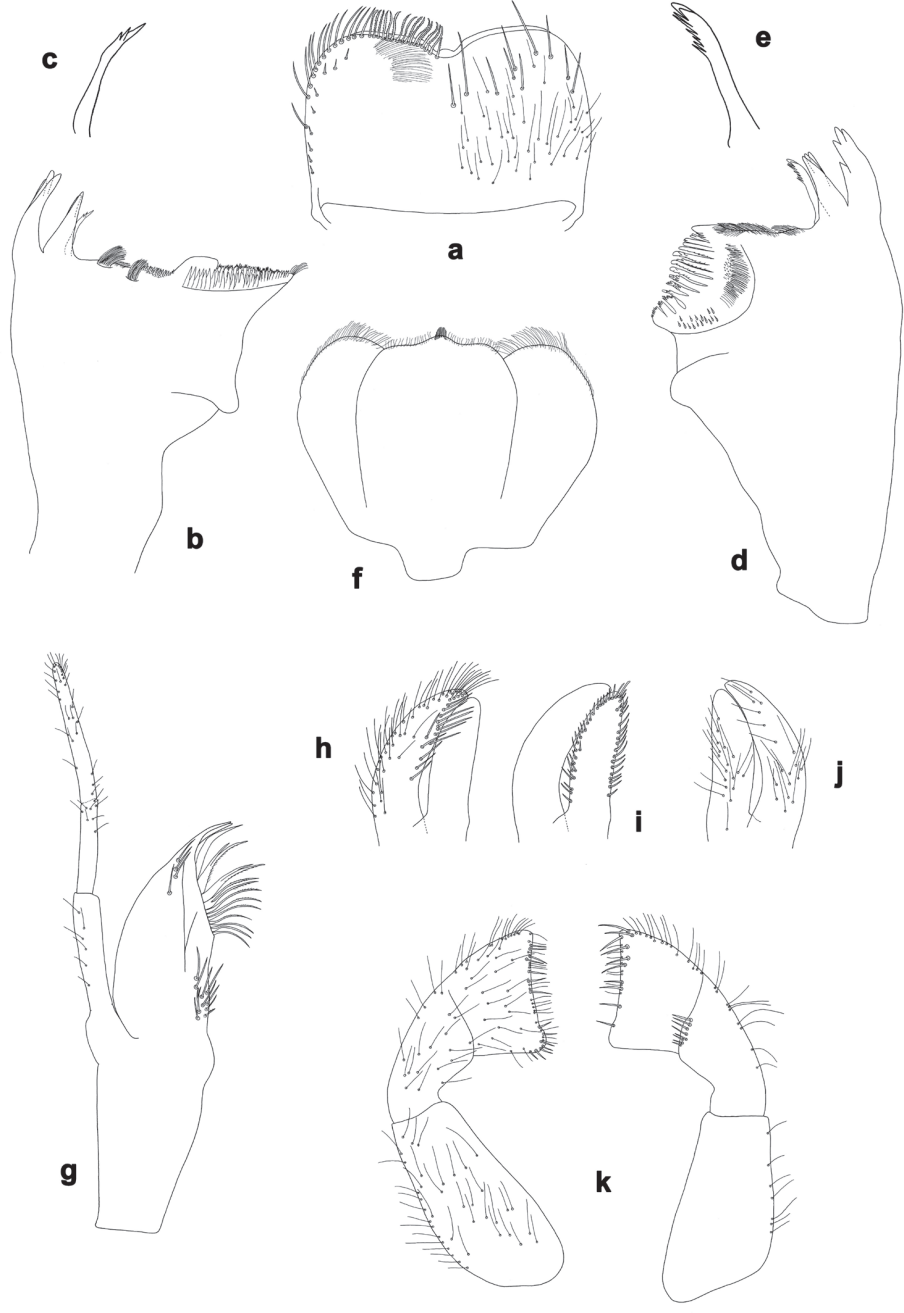
*Centroptilumalamiae* sp. nov., larva morphology **a** labrum (left: ventral view; right: dorsal view) **b** right mandible **c** right prostheca **d** left mandible **e** left prostheca **f** hypopharynx and superlinguae **g** maxilla **h** glossa and paraglossa (ventral view) **i** glossa and paraglossa (ventral view) **j** glossa and paraglossa (dorsal view) **k** labial palp (left: ventral view; right: dorsal view).

**Figure 8.**
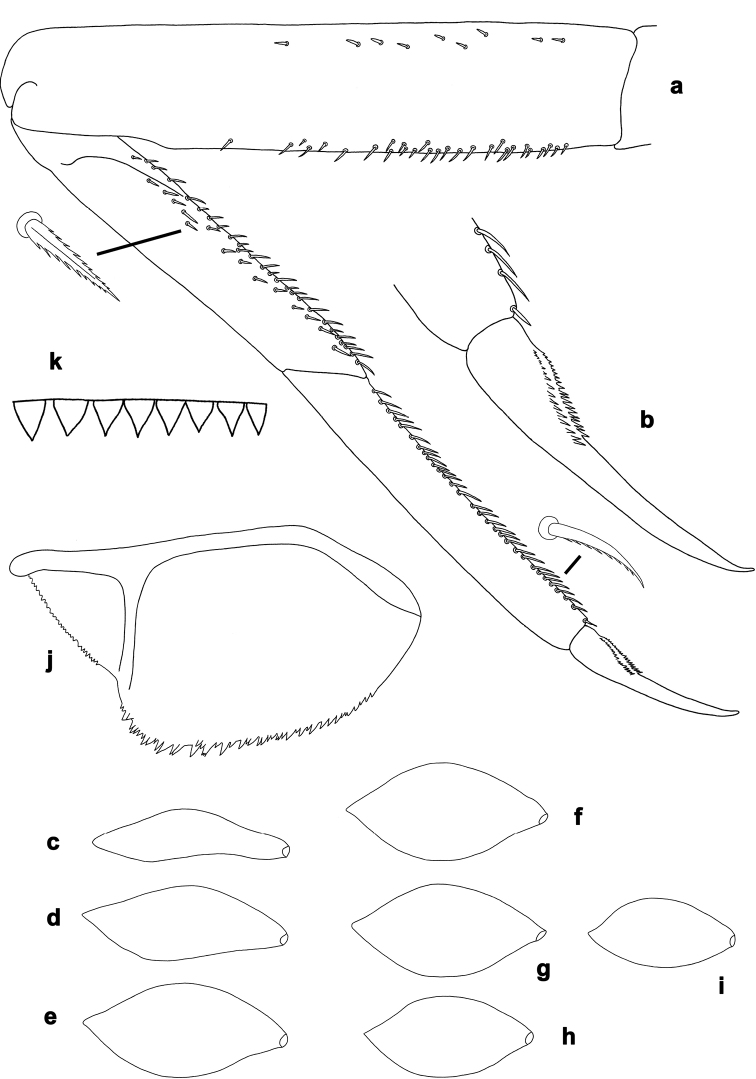
*Centroptilumalamiae* sp. nov., larva morphology **a** foreleg **b** fore claw **c** tergalius I **d** tergalius II **e** tergalius III **f** tergalius IV **g** tergalius V **h** tergalius VI **i** tergalius VII **j** paraproct **k** caudalii, spines on posterior margins of segments.

**Figure 9.**
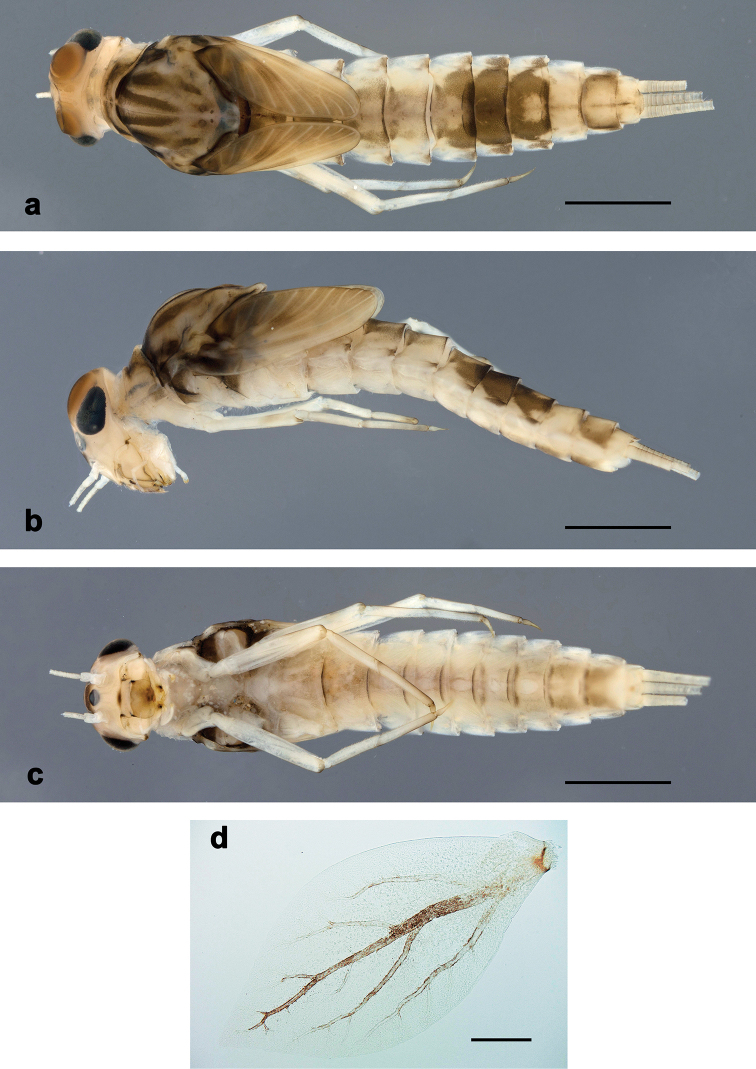
*Centroptilumalamiae* sp. nov., larva **a** habitus, dorsal view **b** habitus, lateral view **c** habitus, ventral view **d** tergalius IV. Scale bars: 1 mm (**a–c**); 0.1 mm (**d**).

Figs 4b, e , 5b , 6 , 7 , 8 , 9

##### Differential diagnosis to other species of *Centroptilum*.

**Larva.** Following combination of characters: A) labrum with anterior margin slightly concave; ratio width vs. length ca. 1.5× (Fig. 7a); B) maxillary palp ca. 1.7× as long as galea-lacinia, segment III apically rounded; segment III ca. 1.6× as long as segment II (Fig. 7g); C) inner distal margin of labial palp segment III slightly concave (Fig. 7k); D) dorsal margin of fore femur with occasional short, spine-like setae; row of stout, pointed setae near margin (Fig. 8a); E) tarsus approx. as long as tibia (Fig. 8a); F) claw with two rows of denticles, each row with ca. 20 small to minute denticles (Fig. 8b); G) paraproct with 30–45 pointed spines, sometimes with split tips, few additional, submarginal spines (Fig. 8j).

##### Description.

**Larva** (Figs 4b, e, 5b, 7–9). Body length 5.6–7.0 mm. Caudalii broken. Antennae reaching apex of fore protoptera.

***Colouration*** (Fig. 9a–c). Head, thorax and abdomen dorsally brown, with dark grey-brown pattern as in Fig. 9a. Head, thorax and abdomen ventrally light brown, with dark grey-brown lateral marks on thorax (Fig. 9c). Legs light brown, femur distomedially slightly darker, tarsus basally and distally slightly darker, claw basally darker. Caudalii light brown, darker annulated.

***Labrum*** (Fig. 7a). Rectangular, width ca. 1.5× maximum length. Distal margin with broad, angulated, medial emargination. Anterior margin slightly concave. Dorsal surface scattered with long, medium and short, simple setae; setae not arranged in a submarginal arc. Ventrally with marginal row of setae composed of anterolateral long, simple, pointed setae and medial long, apically blunt, pectinate setae; ventral surface with ca. nine short, stout setae near lateral and anterolateral margin.

***Right mandible*** (Fig. 7b, c). Incisor and kinetodontium separated. Incisor with three denticles; kinetodontium with two denticles. Prostheca stick-like, distally with three denticles. Margin between prostheca and mola almost straight, with two tufts of long setae. Tuft of setae at apex of mola present.

***Left mandible*** (Fig. 7d, e). Incisor and kinetodontium separated. Incisor with four denticles; kinetodontium with three denticles. Prostheca stick-like, distolaterally denticulate. Margin between prostheca and mola straight, with large brush-like tuft of long setae. Subtriangular process short, on level of area between prostheca and mola. Tuft of setae at apex of mola absent.

***Hypopharynx and superlinguae*** (Fig. 7f). Lingua as long as superlinguae. Lingua longer than broad; distal half laterally not expanded; distal margin with short, fine setae, tuft of stout setae short. Superlinguae distally rounded; lateral margins rounded; fine, short to long, simple setae along distal margin.

***Maxilla*** (Fig. 7g). Galea-lacinia ventrally with four or five simple, apical setae under canines. Canines long and slender. With three denti-setae, distal denti-seta canine-like, middle and proximal denti-setae slender, bifid and pectinate. Medially with one pectinate, spine-like seta and three simple, spine-like setae (dorsolateral insertions); and ca. six long setae, partly with bifurcated tips (bifurcation often difficult to see; ventrolateral insertions). Maxillary palp 3-segmented, ca. 1.7× as long as length of galea-lacinia; palp segment III ca. 1.6× length of segment II; setae on maxillary palp fine, simple, scattered over surface of segments I, II, and III; apex of last segment rounded.

***Labium*** (Fig. 7h–k). Glossa nearly as broad and slightly shorter than paraglossa; inner and outer margins with many short, spine-like setae; apex with two medium, robust setae; dorsal surface with long, fine, simple, scattered setae. Paraglossa curved inward; ventrally with many long setae along outer lateral and apical margin, and row of long, stout, pointed, simple setae along inner lateral margin; dorsal surface with long, fine, simple, scattered setae. Labial palp 3-segmented. Segment III nearly trapezoidal with rounded distal corners, distal margin slightly concave; outer lateral margin with short to medium, fine, simple setae, distal margin with short, spine-like and short, fine, simple setae; ventral surface with medium, fine, simple, scattered setae. Segment II with medium, fine, simple, scattered setae along outer lateral margin and on ventral surface; dorsally with seven or eight short, spine-like setae along distal margin. Segment I with medium, fine, simple setae scattered on ventral surface and on outer lateral margin.

***Hind protoptera*** well developed.

***Foreleg*** (Fig. 8a, b) very slender. Ratio of foreleg segments 1.6:1.0:1.0:0.4. *Femur*. Length ca. 5× maximum width. Dorsal margin with occasional short, spine-like setae, row of short, pointed setae near margin. Apex slightly rounded. Short, stout, pointed setae scattered along ventral margin; femoral patch absent. *Tibia*. Dorsal margin bare. Ventral margin with row of short, curved, spine-like setae and some aditional stout, pointed setae along margin. Anterior surface scattered with short, stout, pointed, and partly serrate setae along ventral margin. Patellatibial suture present in basal 1/3 area. *Tarsus*. Dorsal margin bare. Ventral margin with dense row of short, curved, serrate, spine-like setae. *Claw* with two rows of 17–20 minute denticles each, in basal ca. 1/3 area, increasing in size distally; subapical setae absent.

***Terga*** (Figs 4b, e, 5b). Posterior margin of terga: I smooth, without spines; II–VI (VII) with small triangular spines; VII–IX with triangular, pointed spines.

***Sterna.*** Posterior margin of sterna I–VI smooth, without spines. Posterior margin of sterna VII–VIII with small, triangular spines.

***Tergalii*** (Figs 8c–i, 9d). Present on segments I–VII. Costal margins with minute denticles and short, fine, simple setae, anal margins almost smooth. Tracheae extending from main trunk to inner and outer margins. Tergalius I as long as length of segments II and III combined; tergalius IV as long as length of segments V and VI combined; tergalius VII as long as length of segments VIII and IX combined.

***Paraproct*** (Fig. 8j). With irregular row of 30–45 pointed marginal spines of different size, some with split tips, and few additional spines in second row. Cercotractor with minute, irregular, marginal spines.

***Caudalii*** (Fig. 8k). Spines at posterior margins of segments short triangular, pointed.

**Subimago.** Judging from subimaginal tarsomeres developing under cuticle of last instar female larvae, all tarsomeres of all legs of female subimago have pointed microlepids on surface (see Kluge 2022).

**Imago.** Unknown.

##### Etymology.

Dedicated to Prof. Majida El Alami, committed researcher on aquatic insects in Morocco, and collector of some of the specimens; in recognition of her substantial contribution to the knowledge of the systematics, ecology, and distribution of Moroccan mayflies.

##### Biological aspects.

The specimens were collected in calm edge waters, loose substrate, low to moderate current, high temperatures, and sites rich in filamentous algae and mosses (Fig. 6a, b; El Alami et al. 2022a).

##### Distribution

(Fig. 6e). Morocco.

##### Type-material.

***Holotype*.** Morocco • larva; Oued Kelâa, Akchour; 35°14'32"N, 05°10'10"W; 13.03.2021; leg. S. El Yaagoubi; on slide; GBIFCH00592619, GBIFCH00592620, GBIFCH00592621; MZL. ***Paratypes*.** Morocco • 6 larvae; same data as holotype; 2 on slides; GBIFCH00980875, GBIFCH00980876; 4 in alcohol; GBIFCH00975645, GBIFCH00975646; MZL • 7 larvae; Oued Jnane Niche (sup.); 16.03.2014; leg. M. El Alami; in alcohol; GBIFCH00975647; MZL • 12 larvae; Oued Jnane Niche (sup.); 17.05.2015; leg. M. El Alami; 1 on slide; 11 in alcohol; LESCB.

### ﻿Genetics

**Figure 10.**
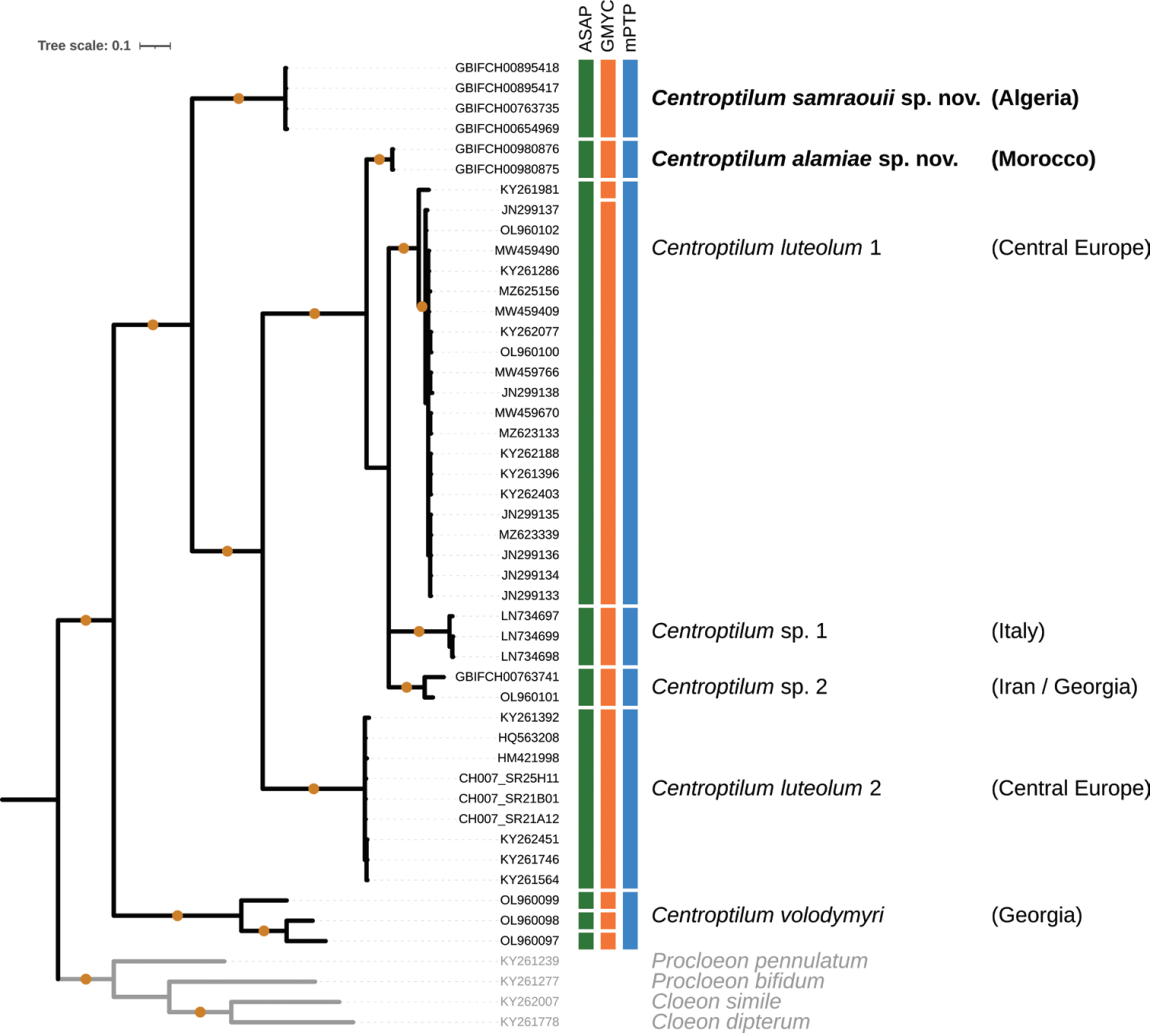
Bayesian majority-rule consensus tree reconstructed from the CO1 data set. Coloured vertical boxes indicate species delimitation hypothesis according to the ASAP, GMYC and mPTP methods. Tips labelled with GBIF codes indicate newly sequenced specimens, CH007_SR codes designate sequences from the FREDIE project, and other codes correspond to previously published GenBank sequences. For each mPTP species hypothesis, the corresponding species names (where available) and the country of origin is provided. Circles on branches indicate Bayesian posterior probabilities > 0.95. Outgroup branches, tips labels, and species names are presented in grey.

The COI ingroup data set was 98% complete and included 34% of parsimony informative sites. The missing data almost exclusively resulted from nine GenBank sequences that lacked 5’ and/or 3’ end. All main CO1 gene tree relationships were resolved and well supported, except for the placement of the three clades *Centroptilum* sp. 1, *C.* sp. 2, and *C.luteolum* 1 (Fig. 10). The four sequences of *C.samraouii* sp. nov. were grouped in a well-supported monophyletic clade, supported as a distinct species in the ASAP, GMYC and mPTP species delimitation analyses (Fig. 10). Similarly, the two sequences of *C.alamiae* sp. nov. were grouped in a well-supported monophyletic clade, supported as a distinct species in all species delimitation analyses. The K2P mean genetic distance within the four *C.samraouii* sp. nov. and the two *C.alamiae* sp. nov. sequences were 0.08% and 0%, respectively. The K2P mean genetic distance between *C.samraouii* sp. nov. and the other six species (or putative species) ranged from 22.1% (mean distance to *C.alamiae* sp. nov.) to 25.2% (mean distance to *C.* sp. 1), whereas it ranged from 9.2% (mean distance to *C.luteolum* 1) to 25.7% (mean distance to *C.volodymyri*) for *C.alamiae* sp. nov. The three species delimitation methods were congruent, except for one slightly divergent sequence within the *C.luteolum* 1 cluster that was isolated by the GMYC, and the three *C.volodymyri* sequences that were all considered as distinct putative species according to ASAP and GMYC.

## ﻿Discussion

### ﻿Differentiating characters between species of *Centroptilum*

The characters differentiating the geographically relatively close species *Centroptilumluteolum*, *C.samraouii* sp. nov. and *C.alamiae* sp. nov. are summarised in Table 2. Most important are the spines on posterior margin of abdominal terga and the spines on paraproct margin (see Table 2). Further reliable characters to differentiate both new species from North Africa are the distal margin of the labrum (straight in *C.samraouii* sp. nov., slightly concave in *C.alamiae* sp. nov.); the distal margin of labial palp segment III (concave in *C.samraouii* sp. nov., slightly concave in *C.alamiae* sp. nov.); the relative length of maxillary palp segment III vs. segment II (1.3× in *C.samraouii* sp. nov., 1.6× in *C.alamiae* sp. nov.); and the setation on dorsal margin of femur (only occasional setae in *C.samraouii* sp. nov., additional row of short, pointed setae near margin in *C.alamiae* sp. nov.) (see Table 2).

**Table 2.** Differentiating characters of new species of *Centroptilum* and *C.luteolum* (Switzerland, VD, Le Chenit, 18 Aug 2001, leg. A. Wagner) (M: 11B and M: 11F refer to figures in Martynov et al. 2022: fig. 11B, F).

Characters	No. in Martynov et al. 2022	*C.luteolum*	Figs	*C.samraouii* sp. nov.	Figs	*C.alamiae* sp. nov.	Figs
**Larva**							
**Head, mouthparts**							
Labrum, width/length ratio	II.1	1.4–1.6		ca. 1.6	1a	ca. 1.5	7a
Labrum, anterior margin	II.3	nearly straight, medial emargination angular		nearly straight, medial emargination angular	1a	slightly concave, medial emargination angular	7a
Maxillary palp, segment III	II.5	(bluntly) pointed apex		pointed apex	1g	bluntly pointed/rounded apex	7g
		ca. 1.2× as long as segment II		ca. 1.3× as long as segment II		ca. 1.6× as long as segment II	
Maxillary palp, length		ca. 1.8× as long as galea-lacinia		ca. 1.9× as long as galea-lacinia	1g	ca. 1.7× as long as galea-lacinia	7g
Rhight mandible, denticles	II.6	3 + 2		3 + 2	1b	3 + 2	7b
Left mandible, denticles	II.7	4 + 2 (rarely 4 + 3)		4 + 3	1d	4 + 3	7d
Labial palp segment III	II.12	Distal (inner) margin concave		Distal (inner) margin concave	1j	Distal (inner) margin slightly concave	7k
**Thorax, legs**							
Legs, colour pattern	III.4	femur with brown band distally; tibia proximally darker		legs light brown; claw darker	3b	femur distomedially darker, tarsus basally and distally darker; claw basally darker	9a–c
Fore femur, dorsal margin	III.6	occasional short, pointed setae on margin		occasional short, pointed setae on margin	2a	occasional short, pointed setae on margin; row of short, pointed, setae near margin	8a
Fore tibia, length vs. tarsus		ca. equal length		slightly longer (ca. 1.1×)	2a	ca. equal length	7a
**Abdomen**							
Terga, posterior margin (spines)	IV.5, 6	I: no spines		I: no spines		I: no spines	
		II–IX: long, narrow triangular, pointed	4c, f	II–III: small triangular	4a, d	II–VI (VII): small triangular	4b, e
			5c	IV–IX: medium triangular	5a	VII–IX: medium triangular	5b
Terga VII–IX, posterolateral part	IV.7	VII: no spines		VII: no spines		VII: no spines	
		VIII: ca. 3 spines		VIII: ca. 5 spines		VIII: ca. 4 spines	
		IX: 10–13 spines		IX: ca. 8 spines		IX: ca. 12 spines	
Sterna, posterior margin (spines)	IV.10	I–IV: no spines		I–VI: no spines		I–V: no spines	
		V: rudimentary spines		VII–IX: very small triangular		VI: rudimentary	
		VI–IX: medium triangular				VII–IX: very small triangular	
Paraproct, distal margin	IV.14	23–30 pointed spines	M: 11B	17–23 pointed spines	2j	30–45 pointed spines	7j
		plus some spines in 2^nd^ row		plus few smaller in 2^nd^ row		partly split tips	
						plus few in 2^nd^ row	
Caudalii, posterior margin of segments (spines)	IV.17	elongated, triangular spines	M: 11F	elongated, triangular spines	2k	triangular spines with	8k
				with long points		short points	

The recently described species *C.volodymyri* (Georgia, Turkey, Iran) differs from *C.samraouii* sp. nov. and *C.alamiae* sp. nov. by several distinct characters: maxillary palp much longer than galea-lacinia (ca. 2.3×); maxillary palp segment I distinctly wider than segment II (only slightly wider in all other species); labrum much wider than long (1.8–2.0×); claw with more than 60 minute denticles in two rows (ca. 30 per row) (Martynov et al. 2022; for respective character states of *C.samraouii* sp. nov. and *C.alamiae* sp. nov. see Table 2).

The poorly known species *C.pirinense* (Pirin Mountains, Bulgaria) differs from *C.samraouii* sp. nov. and *C.alamiae* sp. nov. at least in the very wide labrum (ca. 2.0× wider than long; Martynov et al. 2022: table II), whereas in *C.samraouii* sp. nov. it is ca. 1.6× and in *C.alamiae* sp. nov. ca. 1.5× (see Table 2).

### ﻿Microlepids of subimago

Judging from tarsomeres of subimagos developing under cuticle of female last instar larvae, at least female subimagos of both new species of *Centroptilum* have all their tarsomeres of all legs covered with pointed microlepids. This is in line with *C.luteolum*, which has pointed microlepids on all tarsomeres of all legs of male and female subimagos (Kluge 2022).

### ﻿Genetics and biogeography

The two new North African species described here are highly supported by our CO1-based analyses. First, the minimum mean genetic distance of 9.2% (mean distance between *Centroptilumalamiae* sp. nov. to *C.luteolum* 1) is much higher than the generally accepted intra-/interspecific threshold value of ca. 3% divergence for mayflies (e.g., Ball et al. 2005; Kjærstad et al. 2012; Gattolliat et al. 2015). Second, both new species are well supported in their own monophyletic clade, and third, all three species delimitation analyses are congruent and support the morphological results. Interestingly, the two new species are not supported as closely related, despite their geographical proximity, suggesting a distinct origin. Rather, *C.alamiae* sp. nov., and the European species *C.* sp. 1, *C.* sp. 2, and *C.luteolum* 1 are included in the same well-supported clade sister to the others, which possibly indicates a more recent colonisation event from Europe to Morocco. This hypothesis is supported by the presence of *C.luteolum* 1 in the Pyrenees and in the south of Spain (unpublished sequences from the project FREDIE; not shown in Fig. 10). The type locality of *C.alamiae* sp. nov. in Morocco is geographically closer to the south of Spain than to the type locality of *C.samraouii* sp. nov. in Algeria. All examined specimens of *Centroptilum* in Morocco and Algeria belong to one of the new species and not to *C.luteolum* or any other species of *Centroptilum*. The genus *Centroptilum* seems to be extremely rare in Tunisia, no specimen from this country could be investigated in this study. In conclusion, we cannot formally exclude the presence of *C.luteolum* in the Maghreb at this point in time, but it seems unlikely.

## Supplementary Material

XML Treatment for Centroptilum samraouii

XML Treatment for Centroptilum alamiae

## References

[B1] Ball SL Hebert PD Burian SK Webb JM (2005) Biological identifications of mayflies (Ephemeroptera) using DNA barcodes. Journal of the North American Benthological Society 24(3): 508–524. 10.1899/04-142.1

[B2] Bauernfeind E Soldán T (2012) The Mayflies of Europe (Ephemeroptera). Apollo Books, Ollerup, Denmark, 781 pp. 10.1163/9789004260887

[B3] Benhadji N Hassaine KA Sartori M (2018) *Habrophlebiahassainae*, a new mayfly species (Ephemeroptera: Leptophlebiidae) from North Africa. Zootaxa 4403(3): 557. 10.11646/zootaxa.4403.3.8 29690223

[B4] Benhadji N Sartori M Abdellaoui Hassaine K Gattolliat J-L (2020) Reports of Baetidae (Ephemeroptera) species from Tafna Basin, Algeria and biogeographic affinities revealed by DNA barcoding. Biodiversity Data Journal 8: e55596(14). 10.3897/BDJ.8.e55596 PMC7442755 32879616

[B5] Boumaïza M Thomas AGB (1995) Distribution and ecological limits of Baetidae vs. the other mayfly families in Tunisia: A first evaluation (Insecta, Ephemeroptera). Bulletin de la Société d`Histoire Naturelle 131: 27–33.

[B6] Chakrabarty P Warren M Page LM Baldwin CC (2013) GenSeq: An updated nomenclature and ranking for genetic sequences from type and non-type sources. ZooKeys 346: 29–41. 10.3897/zookeys.346.5753 PMC3821064 24223486

[B7] Chesters D (2013) collapsetypes.pl [computer software]. http://sourceforge.net/projects/collapsetypes/

[B8] Dambri BM Benhadji N Vuataz L Sartori M (2022) *Ecdyonurusaurasius* sp. nov. (Insecta, Ephemeroptera, Heptageniidae, Ecdyonurinae), a new micro-endemic mayfly species from Aurès Mountains (north-eastern Algeria). ZooKeys 1121: 17–37. 10.3897/zookeys.1121.89613 PMC9848610 36760764

[B9] Darriba D Taboada GL Doallo R Posada D (2012) jModelTest 2: More models, new heuristics and parallel computing. Nature Methods 9(8): 772–772. 10.1038/nmeth.2109 PMC4594756 22847109

[B10] El Alami M El Yaagoubi S Gattolliat J-L Sartori M Dakki M (2022a) Diversity and distribution of mayflies from Morocco (Ephemeroptera, Insecta). Diversity 14(6): 498. 10.3390/d14060498

[B11] El Alami M Benlasri M Sartori M Vuataz L Ghamizi M (2022b) A new species of the genus *Prosopistoma* Latreille, 1833 (Ephemeroptera: Prosopistomatidae) from Morocco. ZooKeys 1117: 203–218. 10.3897/zookeys.1117.83539 PMC9848781 36761382

[B12] Ezard T Fujisawa T Barraclough TG (2009) SPLITS: Species’ Limits by Threshold Statistics. R-package. https://rdrr.io/rforge/splits/

[B13] Folmer O Black M Hoeh W Lutz R Vrijenhoek R (1994) DNA primers for amplification of mitochondrial cytochrome c oxidase subunit I from diverse metazoan invertebrates. Molecular Marine Biology and Biotechnology 3: 294–299. http://www.mbari.org/staff/vrijen/PDFS/Folmer_94MMBB.pdf 7881515

[B14] Fujisawa T Barraclough TG (2013) Delimiting species using single-locus data and the Generalized Mixed Yule Coalescent approach: A revised method and evaluation on simulated data sets. Systematic Biology 62(5): 707–724. 10.1093/sysbio/syt033 23681854 PMC3739884

[B15] Gattolliat J-L Cavallo E Vuataz L Sartori M (2015) DNA barcoding of Corsican mayflies (Ephemeroptera) with implications on biogeography, systematics and biodiversity. Arthropod Systematics & Phylogeny 73(1): 3–18.

[B16] Gillies MT (1990) A revision of the African species of *Centroptilum* Eaton (Baetidae, Ephemeroptera). Aquatic Insects 12(2): 97–128. 10.1080/01650429009361395

[B17] Godunko RJ Martynov AV Gattolliat J-L (2018) Redescription of *Nigrobaetisrhithralis* (Soldán & Thomas, 1983) (Ephemeroptera: Baetidae). Zootaxa 4462: 041–072. 10.11646/zootaxa.4462.1.2 30314052

[B18] Hubbard MD (1995) Towards a standard methodology for the description of mayflies (Ephemeroptera). In: Corkum LD Ciborowski JJH (Eds) Current directions in research on Ephemeroptera.Canadian Scholar’s Press, Toronto, 361–369.

[B19] Hurvich CM Tsai CL (1989) Regression and time series model selection in small samples. Biometrika 76(2): 297–307. 10.1093/biomet/76.2.297

[B20] Kaltenbach T Gattolliat J-L (2020) *Labiobaetis* Novikova & Kluge in Borneo (Ephemeroptera, Baetidae). ZooKeys 914: 43–79. 10.3897/zookeys.914.47067 32132855 PMC7046705

[B21] Kaltenbach T Garces JM Gattolliat J-L (2020) The success story of *Labiobaetis* Novikova & Kluge in the Philippines (Ephemeroptera, Baetidae), with description of 18 new species. ZooKeys 1002: 1–114. 10.3897/zookeys.1002.58017 33363429 PMC7746671

[B22] Kapli P Lutteropp S Zhang J Kobert K Pavlidis P Stamatakis A Flouri T (2017) Multi-rate Poisson tree processes for single-locus species delimitation under maximum likelihood and Markov chain Monte Carlo. Bioinformatics 33(11): 1630–1638. 10.1093/bioinformatics/btx025 28108445 PMC5447239

[B23] Katoh K Rozewicki J Yamada KD (2019) MAFFT online service: Multiple sequence alignment, interactive sequence choice and visualization. Briefings in Bioinformatics 20(4): 1160–1166. 10.1093/bib/bbx108 28968734 PMC6781576

[B24] Kechemir LH Sartori M Lounaci A (2020) An unexpected new species of *Habrophlebia* from Algeria (Ephemeroptera, Leptophlebiidae). ZooKeys 953(2): 31–47. 10.3897/zookeys.953.51244 32821194 PMC7398960

[B25] Khadri O El Alami M El Bazi R Slimani M (2017) Ephemeroptera’s diversity and ecology in streams of the ultramafic massif of Beni Bousera and in the adjacent non-ultramafic sites (NW, Morocco). Journal of Materials & Environmental Sciences 8: 3508–3523.

[B26] Kimura M (1980) A simple method for estimating evolutionary rate of base substitutions through comparative studies of nucleotide sequences. Journal of Molecular Evolution 16(2): 111–120. 10.1007/BF01731581 7463489

[B27] Kjærstad G Webb JM Ekrem T (2012) A review of the Ephemeroptera of Finnmark–DNA barcodes identify Holarctic relations. Norwegian Journal of Entomology 59(2): 182–195.

[B28] Kluge NJ (2004) The phylogenetic system of Ephemeroptera. Academic Publishers, Dordrecht, 1–442. 10.1007/978-94-007-0872-3

[B29] Kluge NJ (2012) Non-African representatives of the plesiomorphon Protopatellata (Ephemeroptera: Baetidae). Russian Entomological Journal 20(1): 361–376. 10.15298/rusentj.20.4.02

[B30] Kluge NJ (2016) Redescription of the genus *Cheleocloeon* Wuillot & Gillies, 1993 (Ephemeroptera: Baetidae) with descriptions of three new species from Zambia and Uganda. Zootaxa 4067(2): 135–167. 10.11646/zootaxa.4067.2.2 27395868

[B31] Kluge NJ (2022) Taxonomic significance of microlepides on subimaginal tarsi of Ephemeroptera. Zootaxa 5159(2): 151–186. 10.11646/zootaxa.5159.2.1 36095552

[B32] Kozlov AM Darriba D Flouri T Morel B Stamatakis A (2019) RAxML-NG: A fast, scalable and user-friendly tool for maximum likelihood phylogenetic inference. Bioinformatics 35(21): 4453–4455. 10.1093/bioinformatics/btz305 31070718 PMC6821337

[B33] Kumar S Stecher G Li M Knyaz C Tamura K (2018) MEGA X: Molecular Evolutionary Genetics Analysis across computing platforms. Molecular Biology and Evolution 35(6): 1547–1549. 10.1093/molbev/msy096 29722887 PMC5967553

[B34] Letunic I Bork P (2021) Interactive Tree Of Life (iTOL) v. 5: An online tool for phylogenetic tree display and annotation. Nucleic Acids Research 49(W1): W293–W296. 10.1093/nar/gkab301 PMC8265157 33885785

[B35] Lugo-Ortiz CR McCafferty WP (1997) Contribution to the systematics of the genus *Cheleocloeon* (Ephemeroptera: Baetidae). Entomological News 108(4): 283–289.

[B36] Lugo-Ortiz CR McCafferty WP (1998) The *Centroptiloides* complex of Afrotropical small minnow mayflies (Ephemeroptera: Baetidae). Annals of the Entomological Society of America 91(1): 1–26. 10.1093/aesa/91.1.1

[B37] Mabrouki Y Taybi AF El Alami M Berrahou A (2017) New and interesting data on distribution and ecology of Mayflies from Eastern Morocco (Ephemeroptera). Journal of Materials & Environmental Sciences 8: 2839–2859.

[B38] Martynov AV Palatov DM Gattolliat J-L Bojková J Godunko RJ (2022) Remarkable finding of *Centroptilum* Eaton, 1869 (Ephemeroptera: Baetidae) in Georgia, Turkey and Iran: one new species evidenced by morphology and DNA. The European Zoological Journal 89(1): 827–855. 10.1080/24750263.2022.2090625

[B39] Monaghan MT Wild R Elliot M Fujisawa T Balke M Inward DJ Vogler AP (2009) Accelerated species inventory on Madagascar using coalescent-based models of species delineation. Systematic Biology 58(3): 298–311. 10.1093/sysbio/syp027 20525585

[B40] Pons J Barraclough TG Gomez-Zurita J Cardoso A Daniel PD Hazell S Kamoun S William DS Vogler AP (2006) Sequence-Based Species Delimitation for the DNA Taxonomy of Undescribed Insects. Systematic Biology 55(4): 595–609. 10.1080/10635150600852011 16967577

[B41] Puillandre N Lambert A Brouillet S Achaz G (2012) ABGD, Automatic Barcode Gap Discovery 643 for primary species delimitation. Molecular Ecology 21(8): 1864–1877. 10.1111/j.1365-294X.2011.05239.x 21883587

[B42] Puillandre N Brouillet S Achaz G (2020) ASAP: Assemble species by automatic partitioning. Molecular Ecology Resources 21(2): 609–620. 10.1111/1755-0998.13281 33058550

[B43] R Core Team (2022) R: A language and environment for statistical computing. R Foundation for Statistical Computing, Vienna, Austria. https://www.R-project.org/

[B44] Rambaut A Drummond AJ Xie D Baele G Suchard MA (2018) Posterior summarization in Bayesian phylogenetics using Tracer 1.7. Systematic Biology 67(5): 901–904. 10.1093/sysbio/syy032 29718447 PMC6101584

[B45] Ronquist F Teslenko M Van Der Mark P Ayres DL Darling A Höhna S Larget B Liu L Suchard MA Huelsenbeck JP (2012) MrBayes 3.2: Efficient Bayesian phylogenetic inference and model choice across a large model space. Systematic Biology 61(3): 539–542. 10.1093/sysbio/sys029 22357727 PMC3329765

[B46] Samraoui B Bouhala Z Chakri K Márquez-Rodríguez J Ferreras-Romero M El-Serehy HA Samraoui F Sartori M Gattolliat J-L (2021a) Environmental determinants of mayfly assemblages in the Seybouse River, north-eastern Algeria (Insecta: Ephemeroptera). Biologia 76(8): 2277–2289. 10.1007/s11756-021-00726-9

[B47] Samraoui B Márquez-Rodríguez J Ferreras-Romero M El-Serehy HA Samraoui F Sartori M Gattolliat J-L (2021b) Biogeography, ecology, and conservation of mayfly communities of relict mountain streams, north-eastern Algeria (Insecta: Ephemeroptera). Aquatic Conservation 31(12): 3357–3369. 10.1002/aqc.3719

[B48] Samraoui B Vinçon G Marquez-Rodriguez J El-Serehy HA Ferreras-Romero M Mostefai N Samraoui F (2021c) Stonefly assemblages as indicators of relict North African mountain streams (Plecoptera). Wetlands 41(6): 78. 10.1007/s13157-021-01477-8

[B49] Sanger F Nicklen S Coulson AR (1977) DNA sequencing with chain-terminating inhibitors. Proceedings of the National Academy of Sciences of the United States of America 74(12): 5463–5467. 10.1073/pnas.74.12.5463 271968 PMC431765

[B50] Shorthouse DP (2010) SimpleMappr, an online tool to produce publication-quality point maps. https://www.simplemappr.net [Accessed June 2022]

[B51] Soldán T Godunko R Thomas A (2005) *Baetischelif* n. sp., a new mayfly from Algeria with notes on *B.sinespinosus* Soldán & Thomas, 1983, n. stat. (Ephemeroptera: Baetidae). Genus 16: 155–165.

[B52] Stecher G Tamura K Kumar S (2020) Molecular Evolutionary Genetics Analysis (MEGA) for macOS. Molecular Biology and Evolution 37(4): 1237–1239. 10.1093/molbev/msz312 31904846 PMC7086165

[B53] Suchard MA Lemey P Baele G Ayres DL Drummond AJ Rambaut A (2018) Bayesian phylogenetic and phylodynamic data integration using BEAST 1.10. Virus Evolution 4(1): vey016. 10.1093/ve/vey016 PMC6007674 29942656

[B54] Talavera G Dincă V Vila R (2013) Factors affecting species delimitations with the GMYC model: Insights from a butterfly survey. Methods in Ecology and Evolution 4(12): 1101–1110. 10.1111/2041-210X.12107

[B55] Thomas AGB (1998) A provisional checklist of the mayflies of North Africa (Ephemeroptera). Bulletin de la Société d`Histoire Naturelle 134: 13–20.

[B56] Vuataz L Sartori M Wagner A Monaghan MT (2011) Toward a DNA taxonomy of Alpine *Rhithrogena* (Ephemeroptera: Heptagenidae) using a mixed Yule-Coalescent Analysis of mitochondrial and nuclear DNA. PLoS ONE 6(5): 1–11. 10.1371/journal.pone.0019728 PMC3096624 21611178

[B57] Waterhouse AM Procter JB Martin DMA Clamp M Barton GJ (2009) Jalview Version 2-a multiple sequence alignment editor and analysis workbench. Bioinformatics 25(9): 1189–1191. 10.1093/bioinformatics/btp033 19151095 PMC2672624

[B58] Zerrouk M Dakki M Bennas N El Agbani MA El Alami M Ghamizi M L’Mohdi O Qninba A Himmi O (2021) Nouvelles données sur les macroinvertébrés du Bassin versant du Haut Sebou (Moyen Atlas, Maroc): Insectes, Mollusques et Crustacés. Boletin de la SEA 69: 29–44.

[B59] Zhang J Kapli P Pavlidis P Stamatakis A (2013) A general species delimitation method with applications to phylogenetic placements. Bioinformatics 29(22): 2869–2876. 10.1093/bioinformatics/btt499 23990417 PMC3810850

[B60] Zrelli S Boumaiza M Bejaoui M Gattolliat J-L Sartori M (2011) New reports of mayflies (Insecta: Ephemeroptera) from Tunisia. Revue Suisse de Zoologie 118: 3–10.

[B61] Zrelli S Gattolliat J-L Boumaïza M Thomas A (2012) First record of *Alainitessadati* Thomas, 1994 (Ephemeroptera: Baetidae) in Tunisia, description of the larval stage and ecology. Zootaxa 3497(1): 60. 10.11646/zootaxa.3497.1.6

[B62] Zrelli S Boumaiza M Bejaoui M Gattolliat J-L Sartori M (2016) New data and revision of the Ephemeroptera of Tunisia. Inland Water Biology 3: 99–106.

